# Benefits of Fish Oil Consumption over Other Sources of Lipids on Metabolic Parameters in Obese Rats

**DOI:** 10.3390/nu10010065

**Published:** 2018-01-10

**Authors:** Paula Novato Gondim, Priscila Vieira Rosa, Daniel Okamura, Viviam De Oliveira Silva, Eric Francelino Andrade, Daniel Arrais Biihrer, Luciano José Pereira

**Affiliations:** 1 Department of Veterinary Medicine, Federal University of Lavras (UFLA), Minas Gerais 37200-000, Brazil; pngondim@hotmail.com (P.N.G.); vivian_osbio@yahoo.com.br (V.D.O.S.); ericfrancelinoandrade@gmail.com (E.F.A.); arrais.daniel@gmail.com (D.A.B.); 2 Department of Animal Sciences, Federal University of Lavras (UFLA), Minas Gerais 37200-000, Brazil; priscila@dzo.ufla.br (P.V.R.); danielokamura@hotmail.com (D.O.); 3 Department of Health Sciences, Federal University of Lavras (UFLA), Minas Gerais 37200-000, Brazil

**Keywords:** polyunsaturated fatty acids (PUFA), plasma lipids, dietary fat, *n*-3 fatty acids, metabolic syndrome, *n*-6 fatty acids, saturated fatty acids

## Abstract

This study evaluated the effect of the consumption of different levels and sources of lipids on metabolic parameters of Wistar rats. Animals were fed with high-fat diet (HFD) containing 20% of lard for 12 weeks to cause metabolic obesity. Subsequently, the animals were divided into six groups and were fed diets with lipid concentrations of 5% or 20% of lard (LD), soybean oil (SO) or fish oil (FO), for 4 weeks. Data were submitted to analysis of variance (two-way) followed by Tukey post hoc test (*p* < 0.05). The groups that consumed FO showed less weight gain and lower serum levels of triacylglycerol (TAG), total cholesterol and fractions, aspartate aminotransferase (AST) activity, atherogenic index, less amount of fat in the carcass, decreased Lee index and lower total leukocyte counting (*p* < 0.05). These same parameters were higher in LD treatment (*p* < 0.05). In the concentration of 20%, carcass fat content, blood glucose levels, as well as alanine aminotransferase (ALT) and gamma glutamyl transferase (GGT) decreased in FO groups (*p* < 0.05). The SO group had intermediate results regarding the other two treatments (FO and LD). We concluded that fish oil intake was able to modulate positively the metabolic changes resulting from HFD.

## 1. Introduction

Obesity is a chronic disease [1] with a multifactorial etiology characterized by excessive accumulation of fat in the body [2]. The prevalence of obesity has reached epidemic proportions in many countries. It is considered pandemic and a serious public health problem [3]. In 2014, more than 1.9 billion adults were overweight worldwide and out of these, more than 600 million people were obese [2]. In developing countries, it is estimated that about 52.5% of the population is overweight and 17.9% are obese [4]. Projections for 2022 are that the prevalence of obesity may reach 24.8% [5].

The main cause of excessive accumulation of fat comes from an imbalance between the number of calories consumed and expended [6]. This dysfunction is an important risk factor for the development of comorbidities with high morbidity and mortality, such as cardiovascular disease (CVD), insulin resistance, dyslipidemia, fatty liver, diabetes mellitus, metabolic syndrome [2] and colon, prostate and breast cancer; musculoskeletal (osteoarthritis, risk of fractures, joints injuries) and inflammatory disorders [7], which reduce the quality of life and increase health care costs.

There is evidence that obesity has a positive relationship with lipid composition of the diet. Western diets reflect this, since large amounts of saturated fatty acid (SFA) are consumed along with a high *n*-6:*n*-3 ratio [7,8]. Fatty foods rich in long-chain SFA may contribute to the accumulation of lipids in the body and to dyslipidemias [9,10]. On the other hand, the consumption of unsaturated fatty acids can reduce these changes [11,12]. Among them, the polyunsaturated fatty acids (PUFA) *n*-3 and *n*-6 represented by essential fatty acids (EFA) and its co-products are highlighted. Vegetable oils such as soybean oil are rich sources of *n*-6 PUFA (arachidonic acid-ARA and linoleic acid-LNA) [13], whereas cold-water fishes have great proportions of *n*-3 PUFA (α-linolenic acid-ALA, EPA and DHA) [14].

These EFA cannot be produced in the animal body due to the absence of specific elongases and desaturases enzymes [15]. Therefore, they need to be obtained from their diet. Besides being part of the plasma membranes, EFA play a key role in the homeostasis. LNA is the ARA precursor, essential for the formation of pro-inflammatory eicosanoids. On the other hand, ALA creates the eicosapentaenoic acid (EPA) and docosahexaenoic acid (DHA), which are required in retinal cells, in the brain and which generates mediators with low inflammatory potential [16].

Previous studies have demonstrated the effects of lipogenic *n*-6 and lipolytic *n*-3 [13,17]. The latter reduces insulin resistance [18], inflammatory diseases such as arthritis [7], reduces the risk of CVD, such as atherosclerosis and dyslipidemia involving TAG, total cholesterol and very-long-density lipoprotein (VLDL) [11,19,20].

A better understanding of the metabolic effects of dietary fatty acids (FA) on a daily basis is essential, especially when obesity is already established and people tend to avoid the consumption of oils and fat derivatives. Related experiments usually add lipid sources in the standard diet, however, in the present study, there was a total replacement of dietary lipids to obtain isolated results inherent to the source of lipids without any interference.

We assessed the effect of different quality and quantity of lipid content—lard, soybean and fish oils, in 5% and 20% concentrations—on metabolic parameters of obese rats.

## 2. Materials and Methods

### 2.1. Animals

This study was approved by the Ethics Committee on Animal Use of the Federal University of Lavras (UFLA) under protocol 042/15. All procedures complied with the guidelines of the National Council for Control of Animal Experimentation (CONCEA—SBCAL).

The Central Animal Laboratory of the Federal University of Lavras (UFLA) provided 36 male Wistar rats (Rattus norvegicus albinus) at six weeks of age. The animals were healthy, with initial mean weight of 134.8 g (SD 14.4 g). Animals were distributed and kept in polypropylene boxes under ideal conditions recommended for the species (temperature: 22 ± 2 °C, humidity: 45 ± 15% and light/dark cycle: 12/12 h) throughout the experimental period.

Water and food were provided ad libitum throughout the experimental period. Food and water consumption was assessed daily and body weight measured weekly.

### 2.2. Induction of Obesity

Initially, the animals were randomly divided into groups of six animals per cage and acclimatized to the experimental conditions for one week. Then, animals were fed a hypercaloric HFD, which leads to obesity using a diet containing 20% lard for 12 weeks, as previously described by Araujo et al. [21] (Table 1). Diets were formulated and adapted from the AIN-93 [22].

The chemical composition of the diets was analyzed according to the standard methods established by AOAC (2000) [23].

The addition of 2% soybean oil on diet composition was required to avoid deficiency of EFA in the animals. All the ingredients were mixed, pelleted and frozen. The diet was given daily at room temperature. At the end of the induction period, the Lee index was obtained to confirm obesity (defined by values above 0.300) [24,25].

### 2.3. Experimental Period

**Figure 1:**
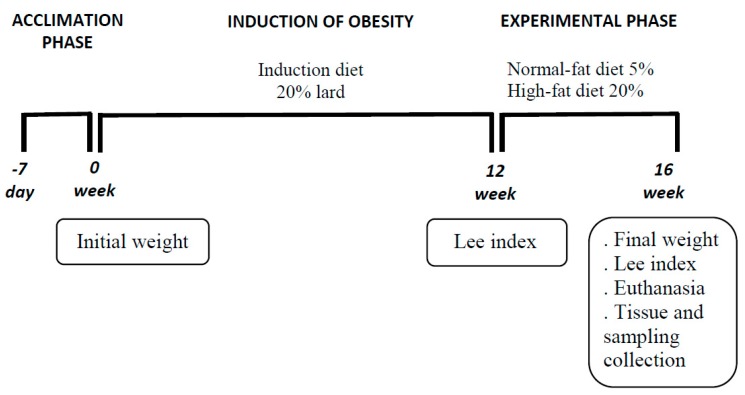
Schematic representation of the experimental design over time.

After obesity was confirmed, the rats were randomly redistributed into six groups of six animals each. The experimental design was completely randomized in a factorial arrangement (3 × 2), consisting of three different lipids added to the diet (lard, soybean oil and fish oil) in two different concentrations: 5% normal-fat diet (NFD) and 20% HFD, comprising six treatments with six replicates each (Table 1). The diets were given for 4 weeks. The fish oil was extracted from salmon and it was acquired from a commercial industry. The diets were prepared weekly to minimize oxidation of the lipid sources. The schematic representation of the experimental design over time is shown in Figure 1.

### 2.4. Fatty Acids Profile

Fatty acids profile of the experimental period diets was estimated based in a previous analysis of experimental diets (Table 2). Briefly, the previous analysis evaluated total lipid content using a modification of the method proposed by Folch, Less and Staney [26]. FA profile was determined using a GC2010 gas chromatograph (GC) (Shimadzu, Kyoto, Japan) equipped with a flame ionization detector and a SP-2560 fused silica capillary column (100.0 m × 0.25 mm, 0.20 µm film; Supleco, Sigma-Aldrich, St. Louis, MO, USA). FA peaks were integrated using a GC solution chromatography software (version 4.02) and peaks were identified by comparison to known standards (37 Component FAME Mix; Supelco, Sigma-Aldrich, Darmstadt, Germany).

### 2.5. Euthanasia and Sampling

At the end of the experimental period, each rat was weighed to calculate the Lee index. Weight gain (difference between the initial and final weight) was determined.

After eight hours of fasting, the animals were sacrificed by cardiac puncture under anesthesia (sodium thiopental 50 mg/kg, ip). Blood samples were collected for biochemical analysis. Whole blood was used for white blood cell count analysis by an automated hematology analyzer SDH-20 (Labtest Diagnostica S/A^®^, Lagoa Santa, MG, Brazil). The serum was used for analysis of the following biochemical parameters: glycemia, total cholesterol, high-density lipoprotein (HDL) cholesterol, TAG, ALT, AST, gamma-glutamyltransferase (GGT) and C-reactive protein (CRP), using commercial specific colorimetric kits (Labtest Diagnostica S/A^®^, Lagoa Santa, MG, Brazil) and specific C-peptide for rats by ELISA (Merck Millipore, Darmstadt, Germany). The levels of low-density lipoprotein (LDL) cholesterol + VLDL-cholesterol fractions from each animal were obtained using the following equation: LDL-C + VLDL-C = total cholesterol—HDL-C [27]. The atherogenic index was calculated according to the relation (VLDL-C + LDL-C)/HDL-C [28].

After blood collection, the animals’ chest and abdominal cavities were opened to expose the internal organs. Heart, liver and right kidney were collected and weighted. Relative organ mass was calculated in relation to the weight of the clean carcass (organ weight/clean carcass weight) [29].

### 2.6. Histological Analysis

For histological analysis, epididymal fat was collected, fixed in 10% buffered formaldehyde and processed routinely for preparation of histological slides with paraffin sections 3 µm thick, stained with hematoxylin and eosin and mounted in resinous Entellan^®^ media (Merck Millipore, Darmstadt, Germany). Histomorphometric analyses were performed using optical microscopy employing a capture system and image analysis, which consists of a binocular Olympus CX31 microscope (Olympus Optical do Brazil Ltda, São Paulo, SP, Brazil) with an attached camera (SC30 CMOS Color Camera for Light Microscopy Olympus Optical do Brazil Ltda, São Paulo, SP, Brazil). Measurements were conducted using Image-Pro^®^ Express software (Targetware Informática Ltda of Brazil, Água Branca, SP, Brazil).

### 2.7. Morphometric Analysis of Adipose Tissue

Epididymal fat cells diameter was determined by measuring the largest distance between two membrane limits in opposite sides [30]. Adipocyte area was determined by measuring the delimitation of the cells and tissue density was calculated using a known squared area superimposed on certain fields of the captured images. Then, the analysis of adipocytes density (number of adipocytes per unit area of tissue) was performed as previously described [31].

All histologic measurements were performed through blind assessment by a single trained evaluator.

### 2.8. Body Composition

Carcasses of animals were weighed, processed and submitted to the analysis of fat and protein using the FoodScanTM NIR (near infra-red) meat analyzer (Foss, Warrington, UK) as performed by Vickers [32]. For carcass analysis, skin, legs, head and viscera were removed.

### 2.9. Data Analysis and Statistics

Data were submitted to two-way analysis of variance (ANOVA) followed by a Tukey post-hoc test using the Sisvar 5.5 Build 82 software (UFLA, Minas Gerais, Brazil) [33] and the results are expressed as means with their standard deviations (SD). A significance level of *p* < 0.05 was used.

## 3. Results

After 12 weeks, all animals fed the 20% lard diet were considered obese (Lee index > 0.300). Subsequently, by the end of the fourth-week of the experimental period, the daily food intake was lower in the HFD groups in relation to NFD. Weight gain of animals was higher in the group that consumed LD than in other groups (Table 3; *p* < 0.05).

Among the NFD groups, results were similar for glycemia. In the HFD groups, animals fed lard showed blood glucose levels 8% higher than SO and 18% higher than FO groups (Table 4; *p* < 0.05). In turn, FO had a reduction of 11% in blood glucose compared to SO. Regarding TAG, a diet rich in FO was effective in reducing this variable, regardless of the concentration (Table 4; *p* < 0.05). Among HFD groups, the LD group had the highest levels of TAG, which were 25% higher than SO and 56% higher than FO (Table 4; *p* < 0.05). Moreover, the SO group was 26% higher than FO. Increased lard content in the LD group diet exacerbated the values of this parameter, from 63 mg/dL to 72 mg/dL (Table 4; *p* < 0.05). Instead, when the FO group content was increased, we observed a more marked reduction of TAG, from 51 mg/dL to 40 mg/dL (Table 4; *p* < 0.05). Regarding C-peptide, the LD 5% group had higher levels compared to FO 5% and among HFD groups, LD was 36.9% higher than SO and 43.7% higher than FO (Table 4; *p* < 0.05).

Regarding serum lipid levels, total cholesterol was lower in the groups receiving FO (Table 5; *p* < 0.05). By increasing the concentration of this lipid to 20%, there was a reduction of about 38% (Table 5; *p* < 0.05). Still regarding this parameter, the LD and SO groups remained equal. In relation to HDL-C among NFD, SO showed higher values than FO (Table 5; *p* < 0.05). However, among HFD, FO had lower levels even when compared with FO 5% (Table 5; *p* < 0.05). The LDL-C + VLDL-C fraction, as well as the atherogenic index followed the same behavior of the total cholesterol (Table 5; *p* < 0.05). At 5% concentrations, among the NFD groups, the atherogenic index for the FO groups was on average 36% lower than other groups (59% lower than the HFD at 20%) (Table 5; *p* < 0.05). When lipid content in the diets increased to 20%, the reduction in the atherogenic index reached 35% (Table 5; *p* < 0.05). 

**Figure 2:**
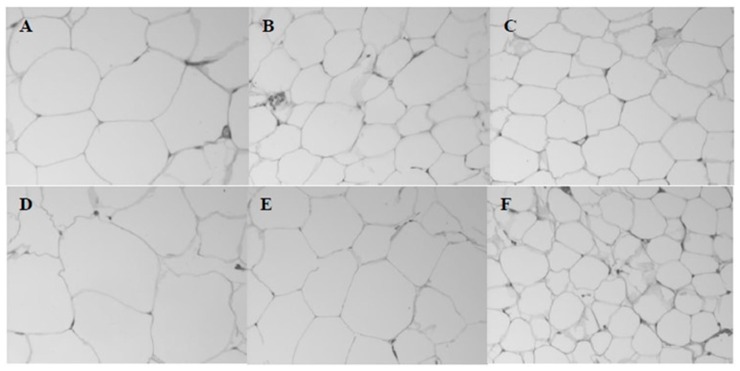
Adipocytes from epididymal fat of obese rats fed diets containing (**A**) 5% lard, (**B**) 5% soybean oil, (**C**) 5% fish oil, (**D**) 20% lard, (**E**) 20% soybean oil and (**F**) 20% fish oil for 4 weeks.

Different lipid sources did not alter the relative weight of the kidney, liver and heart. However, a lower percentage of fat in the carcass was observed in animals fed with FO (Table 6; *p* < 0.05). On the other hand, the protein percentage did not change. The Lee index was also lower in animals fed FO, regardless of the concentration (Table 6; *p* < 0.05). Among HFD, the diameter and the area of adipocytes in the epididymal fat tissue of the FO group was reduced when compared to the LD group (Table 6; *p* < 0.05 and Figure 2). When diets of LD and SO changed to 20%, there was an increase in the diameter and size of adipocytes (Table 6; *p* < 0.05). There were no significant differences for adipocytes density.

The enzymatic activity of AST decreased 8% in the treatment with FO when compared to LD (Table 7; *p* < 0.05). ALT and GGT increased when the lipid concentrations of LD and SO also increased in the diet (Table 7; *p* < 0.05). FO at 20% reduced ALT enzyme activity (35 U/L) compared to LD 20% (46 U/L). Among HFD, GGT values corresponded to the following order: LD > SO > FO (Table 7; *p* < 0.05). CRP did not change between groups but total leukocyte was 21% lower in the group that consumed FO diets (Table 7; *p* < 0.05)

## 4. Discussion

At the end of a 12-week period receiving a high-calorie diet (18.41 kJ/g) (consisting of high fat −20% lard), induction of obesity in the rats was confirmed and the Lee index reached values higher than 0.300 [24,25]. The main objective of this diet was to trigger not just obesity but alterations commonly associated with this pathology such as metabolic syndrome, which was demonstrated in a previous study [21]. High-fat diets are used to induce obesity for the high-calorie and lipogenesis stimulation [9]. Estadella et al. [34] observed that a 30-day fat diet containing 20% of fat was enough to make Wistar rats metabolically obese, with more accumulation of fat in the carcass when compared to animals receiving a standard diet. These authors concluded that the higher the intake period, the more pronounced the weight gain and adiposity.

In this study, food intake was 30% lower in groups that received HFD because they had higher caloric levels compared to NFD. Thereby, a lower intake was sufficient to meet daily total energy requirements [35]. Similarly, Hashimoto et al. [13] observed a reduced consumption among rats receiving HFD, explained by the equivalence of the total energy intake regardless of the type and quantity of food ingested. However, other experiments have shown controversial results such as cafeteria diets [36,37]. Cafeteria diets use palatable food, which stimulates voluntary hyperphagia and, consequently, increase food ingestion, which differs from the present study. Herein, hyperphagia was not promoted by our diet. Therefore, satiety obtained by a high-calorie and HFD was enough to cause a smaller consumption.

Despite the lower consumption, the LD groups had higher weight gain than other diets. This result demonstrates that not only the quantity but also the quality of food intake may contribute to increased body weight [38,39]. Moreover, elevated levels of C-peptide and blood glucose indicated that a lard-rich diet was able to induce insulin resistance in animals. 

Fish oil was able to attenuate obesity regardless of the concentration and it reduced weight gain, leading to Lee index values lower than 0.300. In addition, consumption of this oil was effective in improving metabolic parameters, such as blood glucose, cholesterol and triglycerides and in reducing lipid accumulation in the carcass. These results may be attributed to fatty acids content. The *n*-3 very long-chain (VLC) PUFA acts as an intracellular marker, suppressing the expression of genes involved in lipogenesis and inducing transcription of genes involved in lipid oxidation. By binding to the PPAR and the sterol regulatory element-binding protein (SREBP) nuclear receptors, which function as transcription factors, *n*-3 can regulate the expression of genes involved in glucose and lipid metabolism [40]. This PUFA can increase oxidation of free fatty acids when it activates PPAR-α [41], which in turn acts on the transcription of genes encoding regulatory enzymes of this oxidation [13,42]. The *n*-3 VLC-PUFA also reduces cholesterol biosynthesis by inhibiting the actions of SREBP-1 and SREBP-2 [43,44]. Nakatani et al. [40] found that this modulating effect is dose-dependent, which is consistent with the present study, in which the reduction of serum glucose, TAG and total cholesterol were improved when the FO concentration was increased in the diet. Additionally, Hashimoto et al. [13] suggested that in addition to the inhibition of SREBP-1, the reduction in plasma levels of TAG observed in animals fed with a FO-rich diet was caused by the suppression of Apo-B100 synthesis by the liver and a consequent reduction in the secretion of VLDL. Moreover, we found a reduction of adipocytes’ area and diameter, suggesting the possible lipolytic action induced by *n*-3 PUFA.

A risk indicator for cardiovascular disease is the atherogenic index, which was reduced in the group that consumed FO diets. This index was 35% lower when concentrations of FO were increased to 20%. In this case, the reduction of total cholesterol fractions (HDL, LDL+VLDL) reflects cholesterol lowering as a whole. The atherogenic index becomes important to evaluate the correlation between these fractions, since the higher the LDL (and the lower HDL levels), the higher the risk of atherosclerosis [19,45]. A 59% reduction of this index in the FO group among HFD reflects the beneficial action of *n*-3 in reducing CVD [46].

Animals that consumed SO 5% showed no differences in TAG levels from other NFD groups. However, with the 20% increase of this oil, a difference was evident. From obtained results, we are inclined to believe that Wistar rats have the ability to elongate and desaturate PUFA [47,48,49]. Soybean oil contains an ALA precursor without DHA and EPA derivatives. The increase in soybean oil concentration may increase the amount of ALA necessary for *n*-3 PUFA, EPA and DHA production, which may justify the reduction in TAG levels compared to the lard diet.

Regarding plasma glucose levels among HFD groups, a reduction was observed in the FO group. Studies have shown that saturated fatty acid rich-diets, besides inducing obesity, are able to increase blood glucose levels [34,50] due to the suppression of insulin signaling by TNFα and other inflammatory cytokines, as well as the excess FA, triggering insulin resistance [51,52,53]. Nevertheless, the *n*-3 PUFA is capable of increasing insulin sensitivity by reducing the obesity inflammatory process, as well as the increase in adiponectin levels, which is reduced when there is an excess of adipocytes [54,55].

Decreased plasma activity of ALT, AST and GGT liver enzymes in the HFD/FO group may be associated with its hepatoprotective effect. The measurement of these enzymes is associated with hepatic injury and although they are considered sensitive markers, they have different specificities and half-life time [56]. The accumulation of fat in hepatocytes occurs when the body has reached its maximum deposition capacity and oxidation and this accumulation may trigger hepatic steatosis [10,42].

Different treatments did not affect serum CRP levels, since this parameter corresponds to an acute phase protein [57]. However, the total leukocyte was reduced by an average of 21% in the FO group. This lipid is capable of modulating the inflammatory process initiated by obesity from chemical mediators [58,59,60]. EPA is a substrate for the production of E-series resolvins and DHA can produce D-series resolvins and protectins, which act in the blocking of neutrophil migration, infiltration and recruitment [61]. Besides, EPA is able to synthesize 3 and 5-series eicosanoids that have anti-inflammatory activity [19]. In contrast, 2 and 4-series eicosanoids with pro-inflammatory effects can be produced through ARA after the consumption of soybean oil, as in SFA-rich diets via cyclooxygenase-2 (COX-2) and toll-like receptor-4 (TLR-4) [13]. 

The present study showed that different compositions and concentrations of lipid diets are capable of defining the metabolic profile of rats. Lard is rich in long-chain saturated fatty acids, a fact that exacerbated features of obesity such as weight gain, increased serum levels of glucose, total cholesterol and fractions, TAG and lipid accumulation in the carcass, generating metabolic syndrome. Soybean oil, rich in LNA (*n*-6), kept the metabolic consequences in an intermediate state. Fish oil, rich in *n*-3 PUFA, especially EPA and DHA, was able to reduce these obesity-related metabolic disorders [11,62].

From the analysis, one can assume that the levels and sources of lipids contained in the diet can modulate in different ways obese animal’s metabolism. Therefore, more studies are needed focusing on evaluating the mechanism of action of these FA in relation to hormones, enzymes and nuclear receptors, as well as the ideal lipid concentration, resulting only in beneficial effects.

## Figures and Tables

**Table 1:** Diet ingredients (%) and composition (%) used to induce obesity for 12 weeks and NFD and HFD diets given for four weeks.

	Obesity Induced	NFD			HFD		
		LD 5%	SO 5%	FO 5%	LD 20%	SO 20%	FO 20%
Ingredient							
Cornstarch and sucrose	40.00	55.00	55.00	55.00	40.00	40.00	40.00
Soybean meal (48% protein)	8.60	8.60	8.60	8.60	8.60	8.60	8.60
Casein	20.00	20.00	20.00	20.00	20.00	20.00	20.00
Cellulose	5.00	7.00	7.00	7.00	7.00	7.00	7.00
Lard *	20.00	5.00	0.00	0.00	20.00	0.00	0.00
Soybean oil †	2.00	0.00	5.00	0.00	0.00	20.00	0.00
Fish oil	0.00	0.00	0.00	5.00	0.00	0.00	20.00
Mineral mix	3.00	3.00	3.00	3.00	3.00	3.00	3.00
Vitamin mix	1.00	1.00	1.00	1.00	1.00	1.00	1.00
Choline bitartrate	0.20	0.20	0.20	0.20	0.20	0.20	0.20
Methionine	0.20	0.20	0.20	0.20	0.20	0.20	0.20
BHT	0.02	0.02	0.02	0.02	0.02	0.02	0.02
Vitamin E	0.02	0.02	0.02	0.02	0.02	0.02	0.02
Composition							
Protein	20.91	20.98	21.00	20.87	20.83	20.95	21.01
Energy (kJ/g)	18.41	15.00	15.06	14.99	18.45	18.20	18.39
Fat	22.10	4.99	5.01	5.08	20.09	20.15	20.01
Fiber	5.40	7.38	7.23	7.48	7.37	7.29	7.31
Phosphor	0.20	0.20	0.20	0.20	0.20	0.20	0.20
Calcium	0.20	0.20	0.20	0.20	0.20	0.20	0.20

NFD, normal-fat diet; HFD, high-fat diet; LD, lard; SO, soybean oil; FO, fish oil; BHT, butylhydroxytoluene. * Refined lard (Sadia^®^); † Cargill^®^, São Paulo, SP, Brazil.

**Table 2:** Estimated fatty acids profile (%) of the experimental diets given for four weeks.

Fatty-Acid (%)	NFD			HFD		
	LD 5%	SO 5%	FO 5%	LD 20%	SO 20%	FO 20%
C14:0	0.065	0.023	0.36	0.50	0.03	0.90
C14:1 (*n*-5)	0.00	0.01	0.01	0.00	0.04	0.05
C16:0	15.00	1.94	2.42	60.00	7.74	9.67
C16:1 (*n*-7)	1.00	0.00	0.00	4.00	0.02	0.00
C17:0	0.25	0.02	0.03	1.00	0.09	0.11
C17:1 (*n*-7)	0.15	0.02	0.11	0.60	0.07	0.44
C18:0	6.55	2.62	2.27	26.20	10.48	9.07
C18:1 (*n*-9)	18.55	1.34	0.68	74.20	5.34	2.71
C18:2 (*n*-6)	6.85	4.76	1.26	27.40	19.05	5.04
C18:3 (*n*-3)	0.00	0.75	0.41	0.00	3.02	1.66
C20:0	0.10	0.07	0.00	0.38	0.29	0.00
C20:1 (*n*-9)	0.30	0.06	0.35	1.34	0.29	1.38
C20:2 (*n*-6)	0.25	0.03	0.09	0.90	0.13	0.38
C20:3 (*n*-6)	0.00	0.01	0.04	0.00	0.05	0.14
C20:4 (*n*-6)	0.00	0.03	0.10	0.00	0.14	0.38
C20:5 (*n*-3)	0.00	0.03	0.83	0.00	0.14	3.35
C22:6 (*n*-3)	0.00	0.02	1.27	0.00	0.09	4.90
Total						
Σ SFA	22.40	4.88	5.38	88.60	19.01	20.98
Σ MUFA	20.00	1.43	1.15	79.30	5.26	4.21
Σ PUFA	7.10	5.68	3.95	28.10	22.21	15.32
Σ *n*-3	0.00	0.81	2.48	0.00	3.74	9.45
Σ *n*-6	7.10	4.87	1.48	28.19	19.01	5.58
Σ *n*-9	18.85	1.39	1.03	74.4	5.26	4.33
*n*-3/*n*-6	0.00	0.17	1.68	0.00	0.17	1.68

NFD, normal-fat diet; HFD, high-fat diet; LD, lard; SO, soybean oil; FO, fish oil.

**Table 3:** The daily feed intake (g) and body weight gain (g) of obese rats fed diets containing 5% or 20% lard (LD), soybean oil (SO) or fish oil (FO) in 4 weeks.

%	LD	SO	FO
	Consumption (g/±SD)		
5	25 (3)	28 (3) *	27 (2)
20	20 (2)	18 (1)	18 (3)
	Body weight gain (g/±SD)		
5	324 (5) ^b^	303 (24) ^a^	277 (17) ^a^
20	344 (35) ^b^	285 (28) ^a^	272 (26) ^a^

Values were means (*n* = 6), with their standard deviations (SD). * Differ between the different lipid concentrations by Tukey test (*p* < 0.05). ^a,b^ Followed by different letters differ among diets with the same lipid concentration by Tukey test (*p* < 0.05).

**Table 4:** Parameters related to serum levels of glucose (mg/dL), triacylglycerol (mg/dL) and peptide C of obese rats fed diets containing 5% or 20% lard (LD), soybean oil (SO) or fish oil (FO) in 4 weeks.

%	LD	SO	FO
	Glucose (mg/dL ± SD)		
5	170 (5)	162 (8)	162 (7) ^B^
20	174 (8) ^c^	160 (10) ^b^	143 (7) ^A,a^
	Triacylclycerol (mg/dL ± SD)		
5	63 (2) ^A,b^	55 (10) ^a,b^	51 (9) ^B,a^
20	72 (7) ^B,c^	54 (8) ^b^	40 (6) ^A,a^
	Peptide C (pM ± SD)		
5	496 (94) ^b^	335 (85) ^ab^	223 (89) ^a^
20	490 (165) ^b^	309 (112) ^a^	276 (100) ^a^

Values were means (*n* = 6), with their standard deviations (SD). ^a,b,c^ Followed by different letters differ among diets of the same lipid concentration by Tukey test (*p* < 0.05). ^A,B^ Followed by different letters differ in same diet with different lipid concentrations by Tukey test (*p* < 0.05).

**Table 5:** Parameters related to serum levels of total cholesterol (mg/dL), HDL-cholesterol (mg/dL), LDL + VLDL-cholesterol (mg/dL) and atherogenic index of obese rats fed diets containing 5% or 20% lard (LD), soybean oil (SO) or fish oil (FO) in 4 weeks.

%	LD	SO	FO
	Total cholesterol (mg/dL ± SD)		
5	72 (6) ^b^	73 (8) ^b^	56 (8) ^B,a^
20	71 (5) ^b^	69 (5) ^b^	35 (5) ^A,a^
	HDL cholesterol (mg/dL ± SD)		
5	37 (4) ^a,b^	40 (2) ^b^	35 (4) ^B,a^
20	36 (3) ^b^	37 (2) ^b^	25 (3) ^A,a^
	LDL + VLDL cholesterol (mg/dL ± SD)		
5	35 (5) ^b^	33 (7) ^b^	21 (4) ^B,a^
20	35 (3) ^b^	32 (5) ^b^	10 (4) ^A,a^
	Atherogenic index (±SD)		
5	0.96 (0.17) ^b^	0.83 (0.11) ^b^	0.58 (0.11) ^B,a^
20	0.99 (0.06) ^b^	0.88 (0.07) ^b^	0.38 (0.09) ^A,a^

Values were means (*n* = 6), with their standard deviations (SD). ^a,b^ Followed by different letters differ among diets of the same lipid concentration by Tukey test (*p* < 0.05). ^A,B^ Followed by different letters differ in same diet with different lipid concentrations by Tukey test (*p* < 0.05).

**Table 6:** Parameters related to fat in the carcass (%), Lee index and diameter (µm) and area (μm^2^) of adipocytes from epididymal fat of obese rats fed diets containing 5% or 20% lard (LD), soybean oil (SO) or fish oil (FO) in 4 weeks.

%	LD	SO	FO
	Carcass fat (% ± SD)		
5	9.6 (0.3) ^b^	9.9 (0.7) ^b^	8.2 (1.4) ^a^
20	10.4 (0.8) ^b^	10.1 (0.9) ^b^	9.0 (1.1) ^a^
	Lee index (±SD)		
5	0.309 (0.009) ^b^	0.308 (0.008) ^b^	0.292 (0.010) ^a^
20	0.311 (0.007) ^b^	0.311 (0.005) ^b^	0.296 (0.010) ^a^
	Diameter (µm/±SD)		
5	195 (13) ^A^	183 (7) ^A^	184 (15)
20	210 (9) ^Bb^	200 (9) ^B,a,b^	186 (12) ^a^
	Area (μm^2^ ± SD)		
5	21,797 (3742) ^A^	20,569 (1906) ^A^	22,239 (4310)
20	25,049 (1281) ^B,b^	23,860 (2678) ^B,a,b^	21,080 (1401) ^a^

Values were means (*n* = 6), with their standard deviations (SD). ^a,b^ Followed by different letters differ among diets of the same lipid concentration by Tukey test (*p* < 0.05). ^A,B^ Followed by different letters differ in same diet with different lipid concentrations by Tukey test (*p* < 0.05).

**Table 7:** Parameters related to the enzymatic activity of aspartate aminotransferase-AST (U/L), alanine aminotransferase-ALT (U/L), gamma glutamyltransferase-GGT (U/L) and total leukocyte (10^3^/uL) of obese rats fed diets containing 5% or 20% lard (LD), soybean oil (SO) or fish oil (FO) in 4 weeks.

%	LD	SO	FO
	AST (U/L/±SD)		
5	135 (7) ^b^	132 (12) ^a,b^	123 (4) ^a^
20	144 (9) ^b^	140 (12) ^a,b^	133 (13) ^a^
	ALT (U/L/±SD)		
5	31 (5) ^A^	30 (3) ^A^	32 (3)
20	46 (4) ^B^^,^^b^	41 (5) ^B,a,b^	35 (5) ^a^
	GGT (U/L/±SD)		
5	0.56 (0.07) ^A^	0.55 (0.04) ^A^	0.63 (0.04)
20	2.03 (0.2) ^B,c^	0.91 (0.03) ^B,b^	0.72 (0.08) ^a^
	Total leukocyte (10^3^/uL/±SD)		
5	4.3 (0.1) ^b^	4.2 (0.2) ^b^	3.2 (0.3) ^a^
20	4.0 (0.3) ^b^	4.0 (0.4) ^b^	3.3 (0.2) ^a^

Values were means (*n* = 6) with their standard deviations (SD). ^a,b,c^ Followed by different letters differ among diets of the same lipid concentration by Tukey test (*p* < 0.05). ^A,B^ Followed by different letters differ in same diet with different lipid concentrations by Tukey test (*p* < 0.05).
